# Editorial: The role of the extracellular matrix in tumor progression and therapeutic resistance

**DOI:** 10.3389/fmolb.2022.994506

**Published:** 2022-08-31

**Authors:** Monika Vishnoi, Rohit Upadhyay, William C. Cho

**Affiliations:** ^1^ Department of Neurosurgery, Houston Methodist Research Institute, Houston, TX, United States; ^2^ John W. Deming Department of Medicine, Tulane University School of Medicine, New Orleans, LA, United States; ^3^ Department of Clinical Oncology, Queen Elizabeth Hospital, Kowloon, Hong Kong SAR, China

**Keywords:** extracellular matrix, matrisome, heterogeneity, therapy resistance and tumor progression, cancer

Cancer is the leading cause of death worldwide, with an estimated 19.3 million new cases and 10 million deaths in 2020 (GLOBOCAN 2020) (Sung et al., 2021). Current frontline cancer treatment strategies (targeted therapy and immunotherapy) rely on the genetic information of tumor cells; however, heterogeneous matrix composition, plays a vital role in modulating tumor microenvironment (TME) and developing therapy resistance in individual patients. In 1889, Stephen Paget proposed the “Seed and Soil” theory that described the preference of tumor cells (seed) to grow in an organ-specific microenvironment (matrisome soil) responsible for regulating tumor cellular processes including growth, proliferation, adhesion, migration, and invasion etc. (Mendoza and Khanna, 2009; Langley and Fidler, 2011). In TME, matrisome soil plays an essential role in response to extrinsic/intrinsic cues through the remodeling of extracellular matrix (ECM) mechano-physical properties, stiffness, and reassembling of spatiotemporal architecture affecting metastatic progression, treatment resistance, and disease recurrence (Mendoza and Khanna, 2009; Langley and Fidler, 2011; Cox, 2021; Huang et al., 2021). Thus, deciphering the biology of matrix deposition, decoding environmental cues, and the mechanism of ECM remodeling during cancer progression and metastatic niche development is imperative.

Matrisome/ECM consists of core matrisome (collagens, proteoglycans, and glycoproteins) and associated proteins (matrisome-affiliated, regulatory, and secretory proteins), accounting for 4% of the human proteome (Hynes and Naba, 2012; Socovich and Naba, 2019; Cox, 2021; Huang et al., 2021). ECM proteins are broadly classified into two categories based on their molecular composition: interstitial elements and tissue-specific basement membrane (Cox, 2021; Huang et al., 2021). Aging and anatomic region-dependent environmental cues alter matrix’s biomolecular composition, regulating its spatiotemporal dynamics through reorganizing cytoskeletal elements. During cancer onset, external stimuli trigger aberrant matrix dynamics responsible for overt tumor cell colonization and regional phenotypes. This will lead to intramolecular heterogeneity and treatment resistance in individuals (Hynes and Naba, 2012; Socovich and Naba, 2019; Cox, 2021; Huang et al., 2021). Delineating matrix modulated tumor phenotypes also provides novel insights to determine inter- and intra-patient tumor heterogeneity in precision medicine. Recent advances in high-throughput technologies, single-cell genomics, and spatial biology, have helped us to understand the real-time biology of tumor-matrix development and overcome therapeutic resistance (Bingham et al., 2020). In this Research Topic, we aimed to cover recent advances in matrix biology to understand the mechanism of tumor progression and its role in therapy development. Here, we collected studies providing additional evidence supporting the role of ECM in cancer biology, and focused on developing new prognostic, diagnostic, and therapeutic approaches. A total of 40 international researchers have contributed to a compilation of five articles on the current research topic.

A review article by Fromme and Zigrino provided a comprehensive overview of ECM remodeling during skin cancer progression (Melanoma, cutaneous squamous cell carcinoma, basal cell carcinoma, and Merkel cell carcinoma). The authors summarized different mechanisms of ECM remodeling (collagen deposition, matrix stiffness, etc.) in response to MMPs, MAPK, and checkpoint inhibitors which influence drug efficacy and therapeutic resistance during skin cancer development and progression. Another original research article by Moreira et al. evaluated ECM signatures associated with gastric cancer (GC) progression and identified 142 unique gastric matrisome signatures responsible for the matrix modulation by activating ECM receptors and cellular processes involved in angiogenesis and cell-extrinsic metabolic regulation. Authors identified two prognostic biomarkers lysyl oxidase (LOX) and latent transforming growth factor beta-binding protein 2 (LTBP2), and collagen alpha-1(X) chain (COL10A1) diagnostic marker concordant with the findings of the independent GC cohort available at the TCGA database. King et al. demonstrated the role of LKB1 fibril collagen and glycoprotein matrix modeling in adipocyte-mediated tumorigenesis in triple-negative breast cancer. Furthermore, Jansson et al. studied the prognostic significance of collagen IV in small invasive breast cancers (tumor size ≤ 15 mm). They found a significant correlation between the stromal collagen IV expression with tumor size, age, and distant metastasis in a cohort of breast cancer patients. Future studies focusing on mechanistic validation of these matrix-related biomarkers are imperative in a large patient cohort of different solid cancers. Further matrix interactions with other TME cell types (immune cells, fibroblasts, etc.) will provide greater insights in understanding the role of ECM modeling during cancer progression. This will provide novel ideas to develop new in or combination approaches to improve precise therapy of individuals.

Applying bioinformatic approaches, Tang et al. analyzed a TCGA-LIHC hepatocellular carcinoma database and identified two ECM-related clusters (clusters A and B) based on gene expression profiling. Genomic analysis of these cohorts, revealed that cluster A is associated with high tumor mutational burden, poor response to immunotherapy, worst survival. The authors also observed that cluster A had higher mutation frequencies of *TP53*, *MUC4*, *XIRP2*, *HMCN1*, and *RYR3*, while cluster B had higher mutation frequencies of *IL6ST*, *TRIP12*, and MAP2 genes. Moreover, functional enrichment analysis demonstrated that immune-relevant pathways were significantly enriched in cluster A, while cluster B was significantly enriched with amino acid metabolism-related pathways. Further, expression levels of *SPP1*, *ADAMTS5*, *MMP1*, and *BSG* were associated with poor prognosis of HCC, while *LAMA2* and *CDH1* were associated with prolonged survival. These studies provide further support the notion that targeting the ECM-mediated immunosuppressive microenvironment and physical barriers, along with systemic therapies, could favorably enhance the response to current treatment.

In conclusion, a better understanding of matrisome dynamics during cancer development, progression, and recurrence will provide crucial insights on tumor complexity, cellular heterogeneity, and their response to therapy. This will help us work closer towards personalized therapy for individuals with better outcomes.
